# Characterizing heterogeneity in leukemic cells using single-cell gene expression analysis

**DOI:** 10.1186/s13059-014-0525-9

**Published:** 2014-12-03

**Authors:** Assieh Saadatpour, Guoji Guo, Stuart H Orkin, Guo-Cheng Yuan

**Affiliations:** Department of Biostatistics and Computational Biology, Dana-Farber Cancer Institute, Boston, MA 02215 USA; Department of Biostatistics, Harvard School of Public Health, Boston, MA 02115 USA; Division of Pediatric Hematology/Oncology, Boston Children’s Hospital, Boston, MA 02115 USA; Department of Pediatric Oncology, Dana-Farber Cancer Institute, Boston, MA 02215 USA; Harvard Medical School, Boston, MA 02115 USA; Harvard Stem Cell Institute, Cambridge, MA 02138 USA; Center of Stem Cell and Regenerative Medicine, Zhejiang University, School of Medicine, Hangzhou, 310058 China; Howard Hughes Medical Institute, Boston, MA 02115 USA

## Abstract

**Background:**

A fundamental challenge for cancer therapy is that each tumor contains a highly heterogeneous cell population whose structure and mechanistic underpinnings remain incompletely understood. Recent advances in single-cell gene expression profiling have created new possibilities to characterize this heterogeneity and to dissect the potential intra-cancer cellular hierarchy.

**Results:**

Here, we apply single-cell analysis to systematically characterize the heterogeneity within leukemic cells using the MLL-AF9 driven mouse model of acute myeloid leukemia. We start with fluorescence-activated cell sorting analysis with seven surface markers, and extend by using a multiplexing quantitative polymerase chain reaction approach to assay the transcriptional profile of a panel of 175 carefully selected genes in leukemic cells at the single-cell level. By employing a set of computational tools we find striking heterogeneity within leukemic cells. Mapping to the normal hematopoietic cellular hierarchy identifies two distinct subtypes of leukemic cells; one similar to granulocyte/monocyte progenitors and the other to macrophage and dendritic cells. Further functional experiments suggest that these subtypes differ in proliferation rates and clonal phenotypes. Finally, co-expression network analysis reveals similarities as well as organizational differences between leukemia and normal granulocyte/monocyte progenitor networks.

**Conclusions:**

Overall, our single-cell analysis pinpoints previously uncharacterized heterogeneity within leukemic cells and provides new insights into the molecular signatures of acute myeloid leukemia.

**Electronic supplementary material:**

The online version of this article (doi:10.1186/s13059-014-0525-9) contains supplementary material, which is available to authorized users.

## Background

Characterization of cancer heterogeneity is of immense importance with significant clinical implications. To describe this heterogeneity, a model of considerable current interest posits that tumors are hierarchically organized, and initiated by cancer stem cells, which are able to self-renew as well as to differentiate into all other lineages in the tumor [1].

One of the few cancer-types in which cancer stem cells have been intensively studied is acute myeloid leukemia (AML) [2-4]. AML is a clonal neoplastic disorder that is characterized by an increase in the number of myeloid cells in the bone marrow and an arrest in their maturation, frequently leading to hematopoietic insufficiency [5]. Initial studies showed that only a rare subset of cells have the capacity to initiate the disease upon transplantation and, therefore, have the leukemia stem cell (LSC) property [2]. Further studies suggested that LSCs are located almost exclusively downstream of the normal progenitor compartment based on immunophenotype [6] and that they display a phenotype similar to granulocyte/monocyte progenitors (GMPs) [4]. However, it has also been shown that tumor-initiating activities can be found in immunophenotypically distinct compartments [7]. Therefore, it remains a challenge to dissect the cellular hierarchy within leukemic cells. Similarly, the critical pathways for LSC functions also remain incompletely understood [8-10].

The hematopoietic system is one of the well-studied models for cellular differentiation for which the cellular hierarchy has been characterized [11,12]. The traditional model holds that the self-renewing hematopoietic stem cells (HSCs) are positioned at the apex of the hierarchy and are capable of reconstituting the entire hematopoietic system, through sequential lineage differentiations to multipotent progenitors (MPPs) [13-15], followed by differentiation into common lymphoid progenitors (CLPs) and common myeloid progenitors (CMPs) [16,17]. CMPs can further bifurcate to GMPs and megakaryocyte/erythroid progenitors (MEPs) [18]. However, alternative models for cellular hierarchy have also been proposed [19]. Single-cell analysis further suggests that the CMPs are highly heterogeneous and contain one subgroup that may directly differentiate into megakaryocytes [20].

The recent development of microfluidic-based single-cell sorting technologies [21], high-throughput transcriptomic profiling with a multiplexing quantitative PCR (qPCR) approach [20,22-25] or massively parallel sequencing [26-33], and mass cytometry-based proteomic strategies [34-36] have greatly expanded the capacity for single-cell gene expression profiling, which was traditionally carried out by using fluorescence-activated cell sorting (FACS) with only a few markers, and provided a great opportunity to unearth cellular heterogeneity. These technologies have been used to investigate the development of the normal hematopoietic system, including mapping the cellular hierarchy [20,34], reconstructing transcriptional networks [20,25], and characterizing cellular heterogeneity in other cancers [23,37].

In this paper, we first utilize FACS analysis of seven surface markers and then apply our recently developed multiplexing qPCR approach to systematically investigate the transcriptional profile of 175 genes in 71 leukemic cells in AML. We integrate these data with our previously published dataset on normal hematopoietic cells [20], and utilize an integrated set of computational tools to map the cellular hierarchy within leukemic cells, and to further elucidate the underlying transcriptional networks. Overall, our study provides novel insights into the cellular heterogeneity and organizing principles in AML.

## Results

### Comparing leukemic and normal hematopoietic cells at the single-cell level

**Figure 1:**
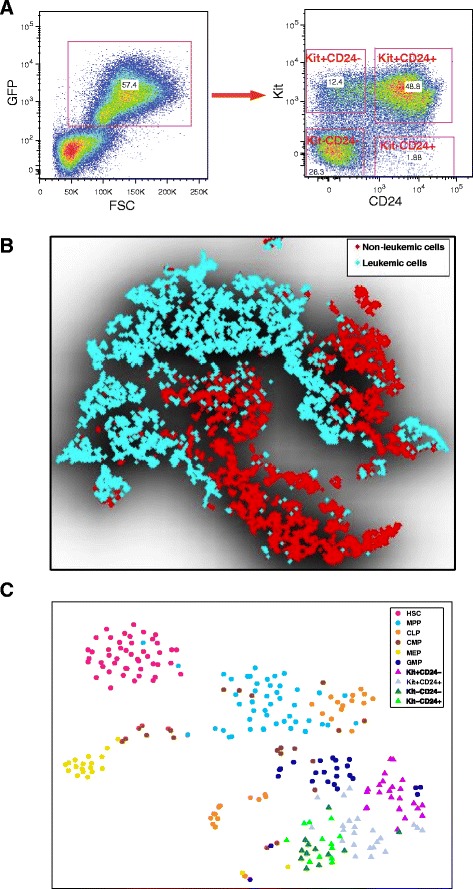
**t-Distributed stochastic neighbor embedding (t-SNE) analysis of FACS and qPCR single-cell gene expression data. (A)** FACS sorting strategy to enrich leukemic and non-leukemic cells. FSC, forward scatter; GFP, green fluorescent protein. **(B)** t-SNE plot of FACS data for a random sample of 5,000 leukemic and 5,000 non-leukemic single cells. Shading shows the outline of the whole data. **(C)** t-SNE plot of the qPCR single-cell gene expression data. Each marker represents a single cell. Leukemic cells are represented by triangles and normal cells are represented by circles. CLP, common lymphoid progenitor; CMP, common myeloid progenitor; GMP, granulocyte/monocyte progenitor, HSC, hematopoietic stem cell; MEP, megakaryocyte/erythroid progenitor; MPP, multipotent progenitor.

Previous studies suggest that the lineage hierarchy in the MLL-AF9 driven leukemia is complex [6,7,20]. Here, we aimed to combine FACS analysis and high-throughput single-cell qPCR analysis to interrogate the differences and similarities between leukemic and normal hematopoiesis. We generated the MLL-AF9 mouse leukemia model using the previously described protocol [8]. We then stained MLL-AF9 primary leukemia bone marrow with antibodies against Flt3, lineage markers (Lin), Sca1, Kit, CD24, CD34, and CD16/CD32, and analyzed the samples by FACS (Figure 1A). These recipient bone marrow cells contain both non-leukemic and leukemic cells. Leukemic cell populations can be distinguished by their green fluorescent protein (GFP) expression, which originates from the MLL-AF9 construct.

Traditional serial two-dimensional gating analysis of the FACS data may introduce bias in defining populations. It is desirable to analyze multiple channels together, integrating information from all seven markers. However, the high dimensionality of the data provides a challenge for visualization. The traditional principal component analysis is ineffective because it relies on a linear assumption, which is violated in single-cell gene expression data. To overcome this limitation, we employed a recently developed nonlinear technique called t-distributed stochastic neighbor embedding (t-SNE) [38], which projects high-dimensional data into a low-dimensional space by converting the Euclidean distances between each pair of data points into heavy-tailed conditional probabilities that represent similarities. The main advantage of t-SNE is that it preserves not only the global layout but also the local structure of the high-dimensional data (see Materials and methods for more details). Similar ideas have been used before to visualize mass cytometry data [39].

t-SNE analysis of the FACS data indicates that the non-leukemic cells are highly heterogeneous (Figure 1B; Additional file 1). Notably, the leukemic cells display much stronger heterogeneity compared with non-leukemic cells. In addition, there is strong overlap between leukemic and non-leukemic cells, suggesting that there remains a high degree of similarity between them, possibly due to incomplete cell-fate transitions, and that these cells are difficult to separate based on immunophenotyping alone.

One limitation of the FACS technique is that only a small number of markers can be simultaneously profiled due to spectral overlapping. In previous work, we developed and optimized a microfluidic-based multiplexing qPCR strategy to accurately profile the gene expression levels in more than 1,000 normal hematopoietic cells extracted from wild-type mouse bone marrow [20]. Here, we applied a similar strategy to systematically investigate the transcriptomic diversity within leukemic cells. In order to explore potential molecular mechanisms underlying lineage specification, we assayed the expression levels of 175 carefully selected genes, including lineage-specific transcription factors, epigenetic modifiers, and cell-cycle regulators, in 71 individual leukemic cells (Additional file 2). In order to include cells from different leukemic cell lineages and to enrich progenitor leukemic cell populations, we used FACS to select four groups of leukemic cells, corresponding to Kit+CD24- (24), Kit+CD24+ (23), Kit-CD24- (12), and Kit-CD24+ (12), where the number of cells in each group is given in parentheses. For comparison, we used the expression levels of the same set of genes in 190 normal cells in wild-type mice as control [20].

As an initial glimpse of the transcriptome landscape, we applied the t-SNE method to project the qPCR data onto a two-dimensional plane (Figure 1C). The color-coding was overlaid on the t-SNE map to help better visualize different cell types. The positions of the HSCs and normal progenitor cells are consistent with their lineage relationships. In particular, HSCs form a clearly defined cluster, whereas MPPs are positioned between HSCs and more specialized progenitors. Of note, the leukemic cells are positioned proximal to GMPs but distal to HSCs.

**Figure 2:**
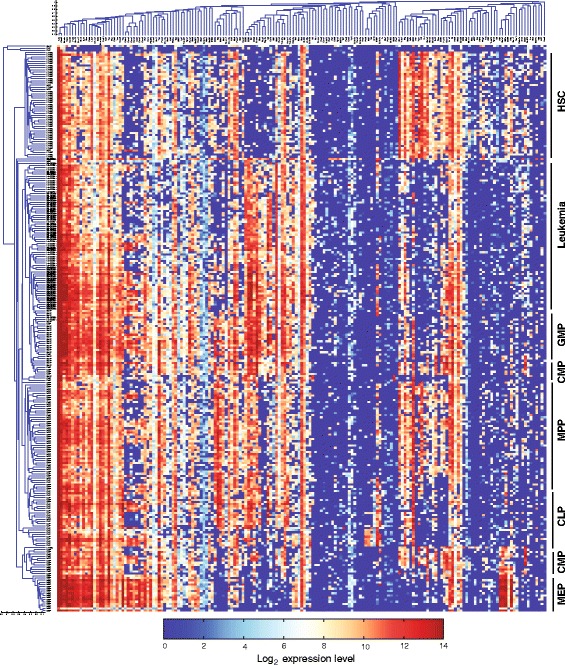
**Hierarchical clustering of the single-cell gene expression data.** Each column corresponds to a specific gene and each row corresponds to a single cell. A representative cell lineage for each cell cluster is shown on the right. CLP, common lymphoid progenitor; CMP, common myeloid progenitor; GMP, granulocyte/monocyte progenitor; HSC, hematopoietic stem cell; MEP, megakaryocyte/erythroid progenitor; MPP, multipotent progenitor.

Unsupervised hierarchical clustering according to the single-cell gene expression profile correctly positioned the normal cells of common lineages next to each other (Figure 2), indicating the high quality of the data. An interesting exception is that CMPs form two separate clusters, which are positioned next to GMPs and MEPs, respectively. Such heterogeneity among CMPs is consistent with our previous study, which further showed that these two subgroups are primed to different cell lineages [20]. In addition, the leukemic cells form a distinct cluster next to GMPs. The leukemic cells express a number of GMP-specific genes, including *CD48*, *CD52*, *CD53*, *Sell*, *Cebpa*, and *Dtx4*, but not the key HSC-specific genes such as *Gata2*, *Hlf*, and *Mpl*, suggesting that leukemic cells resemble a GMP-like cell state but are highly distinguishable from HSCs. Similar results were obtained by using a self-organizing map [40] (data not shown). This is consistent with a model in which leukemic cells originated from GMPs [4].

### Mapping cellular hierarchy identifies subtypes of leukemic cells

In order to map the cellular hierarchy in leukemic cells, we took advantage of a reference map recently identified in the normal hematopoietic system by using single-cell analysis [20]. This map was obtained by profiling the expression levels of commonly used cell surface markers (280 genes) in more than 1,000 cells followed by construction of a minimum spanning tree using the SPADE (spanning-tree progression analysis of density-normalized events) algorithm [34,41]. Each branch of the tree represents a group of cells with similar lineage relationships. In order to map each leukemic cell to the SPADE tree, we implemented a strategy using information only from the set of 33 genes (Additional file 3) profiled in both datasets. To test whether these 33 common genes were sufficient for reproducing the cellular hierarchy, we first applied this strategy to re-analyze the original dataset in [20]. We calculated the mean expression profile of these 33 genes for each node in the SPADE tree, and its Euclidian distance to each cell in the dataset. Then a cell was mapped to the node corresponding to the smallest distance. While the SPADE tree was originally constructed by using information from all 280 genes, we found that our mapping strategy preserves the essential lineage relationships. In total, 90% of the cells were projected to the proximity (≤2 steps) of their original position in the SPADE tree, and 63% of the cells were mapped exactly to the same node (Figure S2A in Additional file 4). For comparison, we randomly selected 100 gene lists, each containing 33 genes, and examined how well the lineage relationships are preserved. We found that, on average, 84% of the cells were mapped to the proximity (≤2 steps) of their original position in the SPADE tree (Figure S2B in Additional file 4), suggesting that the mapping accuracy can be largely preserved by the use of a relatively small number of genes.

**Figure 3:**
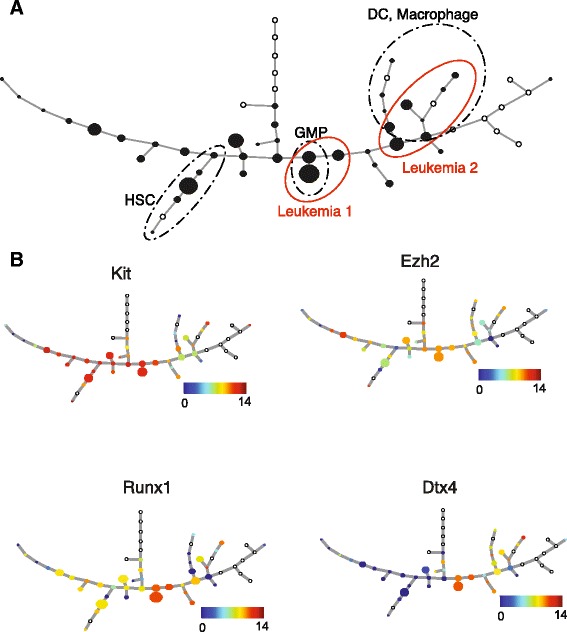
**Mapping cellular hierarchy using SPADE analysis. (A)** The cellular hierarchy in the normal hematopoietic system is represented by the SPADE tree (adapted from [20]), on which the leukemic and normal cells are mapped as described in the main text. Open circles denote clusters to which no cells were mapped. The size of each non-empty node is scaled according to the associated number of cells. The branches corresponding to HSC, GMP, and dendritic cell (DC)/macrophage lineages according to [20] are labeled. **(B)** The mean expression levels for four select genes are overlaid on the SPADE tree.

We next applied this strategy to map our leukemic and control cells to the SPADE-derived cellular hierarchy (Figure 3A). Again, most normal cells are mapped to the expected branches, whereas the cells that are mapped to a different location might result from imperfect clustering. Notably, the leukemic cells are further divided into two subgroups, each projected onto a separate branch of the SPADE tree. The first group (which we call Leukemia 1, containing 29 cells) is mapped to a branch corresponding to GMPs, whereas the second group (which we call Leukemia 2, containing 42 cells) is mapped to the branch corresponding to dendritic cells/macrophages. In addition to the 33 common genes, using the expression profile of about 140 additional genes in our dataset (Additional file 2) provides an opportunity to uncover important differences between the leukemic cells and their closest normal lineages. We used the Wilcoxon-Mann-Whitney rank sum test [42], a robust and non-parametric method, to compare the gene expression levels between Leukemia 1 cells and GMPs. We used a stringent criterion (adjusted *P*-value <1E-5 and absolute log fold change ≥2) for differential gene expression in order to enhance specificity. We note that while we may miss certain genes that are differentially expressed between the two populations, the advantage of choosing a stringent cutoff is that we can then focus on the genes that truly differ between the two cell types. Using this cutoff, we identified four differentially expressed genes, namely *Meis1*, *Cdkn2c*, *Pecam1*, and *Aebp2* (Additional file 5, which also includes adjusted *P*-values for the rest of the genes). The most differentially expressed gene is *Meis1* (log fold change = 7.77), consistent with the previous finding that *Meis1* is an essential and rate-limiting regulator of MLL-induced LSC potential [43]. The fact that *Meis1* is highly expressed in Leukemia 1 suggests that this subgroup of cells may be highly aggressive. Interestingly, *Cdkn2c*, a negative regulator of cell cycle, is also over-expressed in Leukemia 1 cells.

We next compared the transcriptional profiles between the two subgroups of leukemic cells and identified 14 differentially expressed genes (adjusted *P*-value <1E-5 and absolute log fold change ≥2), all of which are up-regulated in Leukemia 1 (Additional file 5). The mean expression levels for four such genes are overlaid on the SPADE tree in Figure 3B. Among the differentially expressed genes, *Kit* is a well-characterized marker, which is frequently mutated in AML. *Etv6* and *Runx1* are known leukemic regulators [44-46], and *Suz12* and *Ezh2* are core members of the Polycomb repressive complex 2 (PRC2), whose activities have been shown to be essential for MLL-AF9 driven leukemia [8]. In particular, *Ezh2* inhibition was recently found as a therapeutic strategy for *Ezh2*-mutant cancers [47,48]. Furthermore, *Brd3* is a member of the bromodomain-containing protein family associated with wide-range activation of super-enhancers in cancer [49].

**Figure 4:**
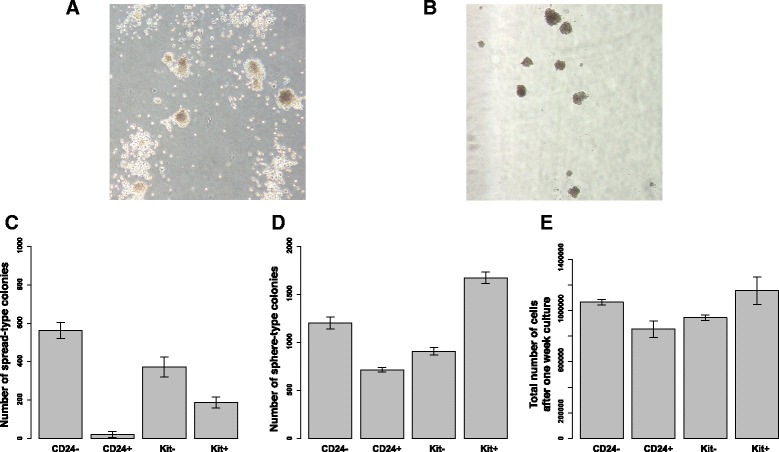
**Experimental validation of the functional difference between the two leukemic cell subtypes. (A)** Snapshot of a colony-forming assay of CD24*-* leukemic cells on day 7. **(B)** Snapshot of a colony-forming assay of CD24*+* leukemic cells on day 7. **(C)** Number of spread-type colonies of different leukemic cell subpopulations. **(D)** Number of sphere-type colonies of different leukemic cell subpopulations. **(E)** Total number of cells after one-week culture in methylcellulose supplemented with IL3, IL6, IL7 and stem cell factor. The error bars represent standard deviation.

We then aimed to test whether these subtypes of leukemic cells have different functions. However, one challenge was that only a few of the differentially expressed genes were surface markers, making it difficult to purify each population *a priori*. Nonetheless, we recognized that *Kit* was the most differentially expressed gene between the two cell types (adjusted *P*-value = 7.91E-11; Additional file 5), and that 83% of the leukemic cells with Kit+CD24- immunophenotype were mapped to Leukemia 1 as opposed to Leukemia 2 cells. Therefore, we FACS sorted subpopulations in the primary leukemia using *Kit* and *CD24* markers as a proxy to the two leukemic cell subtypes, with the caveat that these two markers are insufficient to completely distinguish the two subtypes. We performed *in vitro* colony-forming assays to test proliferation rate and differentiation capability of these sorted cells. Our results indicate that differential expression of these markers correlates with different clonal activity (Figure 4). In particular, we observed that CD24- leukemic cells grew much faster and generated both adhesive sphere-type colonies and non-adhesive spread-type colonies. On the other hand, CD24+ leukemic cells generated significantly fewer colonies (predominantly adhesive sphere-type colonies). This is consistent with previous *in vivo* transplantation experiments, suggesting that CD24- leukemic granulocyte-monocyte progenitors (LGMPs) are more potent for inducing leukemia than CD24+ LGMPs [20]. Furthermore, Kit+ leukemic cells generated a greater number of sphere-type colonies than the Kit- leukemic cells. We note that in Leukemia 1 cells, 72% of the population have a CD24- immunophenotype and 97% of the population have a Kit+ immunophenotype. Therefore, our experimental data provide further evidence that Leukemia 1 cells are more proliferative than Leukemia 2 cells.

### The leukemic cell subtypes are characterized by distinct co-expression networks

**Figure 5:**
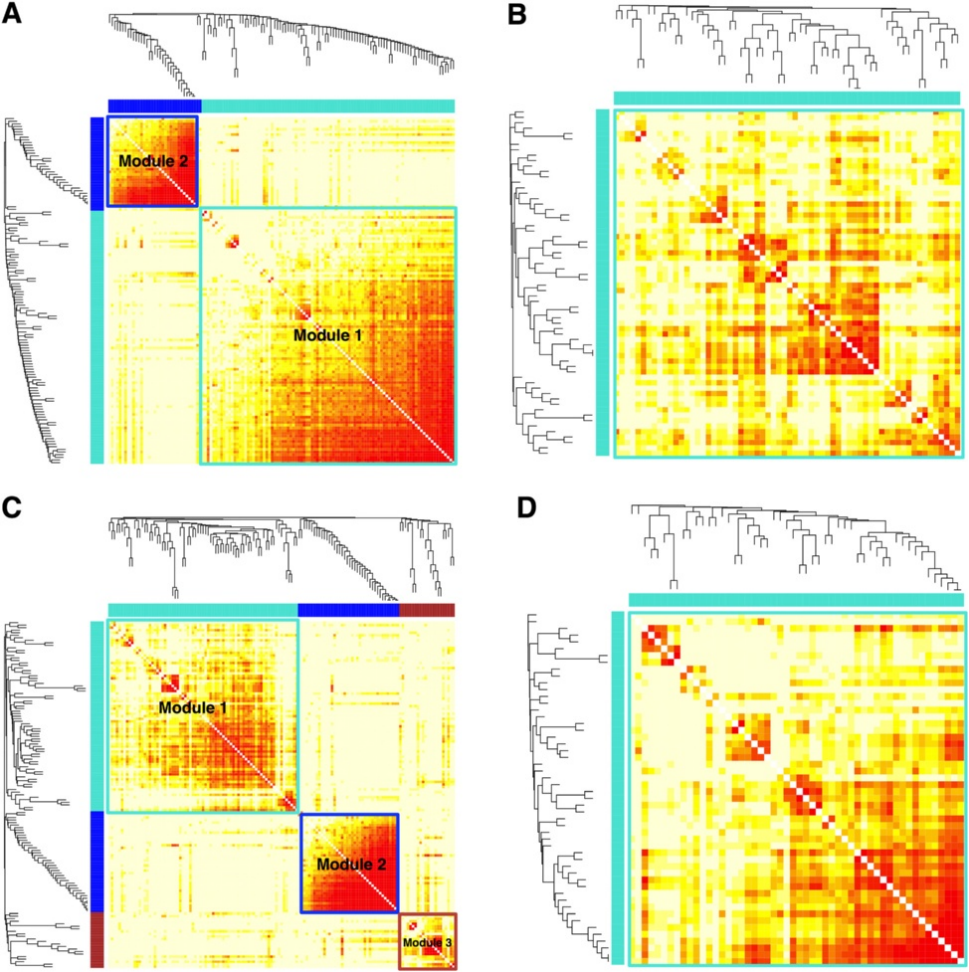
**Network modules identified by weighted gene co-expression network analysis.** Co-expression networks for: **(A)** all leukemic cells; **(B)** GMPs; **(C)** Leukemia 1 cells; and **(D)** Leukemia 2 cells. Each row and column corresponds to a gene. The modules are indicated by the color bars next to the heat map. Light color in the heat map indicates low topological overlap and progressively darker red represents higher topological overlap.

Genes do not function independently but rather interact in concert through a complex regulatory network. In order to systematically identify gene modules with coordinated activities in leukemic cells at the single-cell resolution, we employed weighted gene co-expression network analysis (WGCNA) [50,51]. By analyzing one subset of cells at a time, we constructed four co-expression networks, corresponding to GMPs, all leukemic cells, Leukemia 1 cells, and Leukemia 2 cells (Figure 5). Both all-leukemia and Leukemia 1 networks are further divided into multiple modules containing highly correlated (or anti-correlated) genes, whereas the GMP and Leukemia 2 networks each contains a single module. The list of genes in each module is given in Additional file 6.

Consistent with our t-SNE and hierarchical clustering analyses, the GMP and all-leukemia networks share significant similarity. Of the 64 genes in the GMP module, 56 (88%) are contained in Module 1 of the all-leukemia network (Additional file 7). Surprisingly, three of the differentially expressed genes (*Meis1*, *Cdkn2c*, and *Aebp2*) are contained in this common module, suggesting that cell-fate differences largely reflect different states of a common regulatory circuitry. To see that this is not a contradiction, we note that differential expression and differential co-expression are two distinct modes of changes. Differential expression reflects the change of activity of a gene in isolation whereas differential co-expression reflects the change of correlation between a pair of genes. As a simple example, if the expression level of transcription factor *A* is higher in condition 1 compared with condition 2, and that gene *B* is a direct target of *A* whose expression level is positively correlated with *A*, then both *A* and *B* are differentially expressed between the two conditions, but they are not differentially co-expressed, since their relationship remains the same. Generally speaking, if a set of genes is co-regulated, then differential expression may occur without change of co-expression.

We also found important differences between the GMP and all-leukemia networks. Among the 39 genes that are uniquely contained in Module 1 of the all-leukemia network (Additional files 6 and 7) are a number of well-characterized oncogenes, such as *Ar*, *Bmi1*, *ETS1*, *Kit*, *Lin28a*, as well as tumor repressor genes, such as *Rb1*. The all-leukemia network also contains an additional module, Module 2, which is likely to be regulated independently of Module 1. Module 2 has little overlap (2/35) with the GMP network (Additional file 7). Out of 35 genes in Module 2, 14 (Additional files 6 and 7) are associated with regulation (either positively or negatively) of cell proliferation, including *Bcl11b*, *Flt3*, *Gata3*, *Cdkn1a*, *Cdkn2a*, *Tek*, *Esr1*, *Pbx1*, *Cdkn2b*, *APC*, *Tcf7*, *Tgfb2*, *Tgfb3*, and *Mycn*. Among these genes, *Pbx1* is especially important because it has been shown to be a critical gene required in leukemia initiation [52,53].

We next compared the Leukemia 1 and Leukemia 2 networks to identify subtype-dependent differences. These two networks have strikingly different modular structures. The Leukemia 1 network contains three modules, although Module 3 is rather small and less organized; whereas the Leukemia 2 network contains only a single module. Of the 52 genes in the Leukemia 2 network, 31 (60%) are common with Module 1 of the Leukemia 1 network (Additional file 7). This common set contains 5 of the 14 genes that are differentially expressed between these two subtypes, further suggesting that differential gene activities are coordinately regulated through a common circuitry. This conclusion does not depend on the exact *P*-value cutoff for differential expression, as we arrive at similar results by choosing the top 30 or 50 differentially expressed genes between the two leukemic cell subtypes (that is, a 2.1- or 3.6-fold increase in the number of differential genes, respectively). In these cases, we found a 2.4- or 3.6-fold increase in the number of differentially expressed genes that belong to the common module of the two subtypes. This approximately linear relationship suggests that our conclusion is unaffected by the number of genes in question. We note that only 5 of 40 genes in Module 2 of the Leukemia 1 network are shared in the Leukemia 2 network (Additional file 7). Comparison with the all-leukemia network suggests that Module 2 of the all-leukemia network is retained in Module 2 of the Leukemia 1 network, sharing 33 genes, but lost in the Leukemia 2 network (Additional file 7). Similar results were obtained by using an alternative approach called DiffCoEx [54], which systematically identifies differentially co-expressed modules between two conditions by grouping genes according to their shared, but subtle, differential correlation patterns (see Additional files 8, 9, and 10 for details). Taken together, these analyses suggest that there are significant network differences between the leukemic cell subtypes.

## Discussion

Cancers are associated with distinct heterogeneity. Molecular characterization and functional analysis of such heterogeneity are critical for understanding their origin and progression and treatment outcomes, which may then serve as an important guide for developing new therapeutic strategies. Here, we applied single-cell gene expression analysis to systematically characterize the cellular heterogeneity in AML using an MLL-AF9 driven mouse model. Our analysis identified significant variation of gene expression profiles within the leukemic cells, which can be explained in part by the differences in their corresponding gene networks.

The t-SNE analysis and unsupervised hierarchical clustering suggest that the transcriptomic state of leukemic cells is close to GMPs and far away from HSCs, supporting a model in which LSCs are not directly linked with HSCs [4]. We further investigated the cellular hierarchy by using a previously generated lineage tree of the normal hematopoietic system as a guide [20]. We found that the mapping accuracy was quite high even with a relatively small number of markers (33 genes common between the genes in Additional file 2 and the dataset used in [20]), suggesting significant redundancy among the cell surface markers. The robustness of this strategy suggests that it may have potential applications in mapping cellular hierarchy of other single-cell data in the hematopoietic system. Using this mapping strategy, we found two subtypes of leukemic cells with one (Leukemia 1) resembling GMPs and the other (Leukemia 2) resembling macrophage and dendritic cells. However, differences still exist between Leukemia 1 cells and normal GMPs. Importantly, *Meis1*, a rate-limiting factor for the development of AF9-MLL induced AML [43], is highly expressed only in the leukemic cells. We also found important differences between the two subtypes of leukemic cells, with Leukemia 1 cells overexpressing a number of important leukemia regulators, including *Etv6* and *Runx1*, providing support that these cells are more important for tumor initiation. Notably, Leukemia 1 cells also over-express a number of chromatin regulators, including *Brd3* and Polycomb complex members *Ezh2* and *Suz12*, all of which have been linked with leukemia and other cancers [8,47,48]. By using *in vitro* colony-forming assays we found that the Leukemia 1 population, which is enriched with a Kit+CD24- immunophenotype, has a higher proliferation rate and differentiation capability than the Leukemia 2 population. However, we note that *Kit* and *CD24* markers alone are insufficient to completely distinguish the two leukemic cell subtypes.

Network modeling is increasingly recognized as a powerful tool for understanding complex biological systems, including the hematopoietic system [55,56]. Efforts are underway to apply network-modeling approaches for the computational elucidation and analysis of single-cell data [20,25,30]. Here, we employed a co-expression network-based method (WGCNA, [50,51]) to analyze single-cell gene expression data, using the identified cellular hierarchy as a guide. Our analysis identified a core module that is common between GMPs and leukemia networks, and suggested that much of the gene expression level changes between these two cell types can be viewed as a switch of allowable states within a common network module. On the other hand, we also identified significant differences between the networks. For example, *Pbx1*, which cooperates with *Meis1* in leukemogenesis [53], is regulated by a separate module. As such, our analysis demonstrates that network modeling provides mechanistic insights into organizing principles of leukemia.

LSCs are associated with poor prognosis and treatment failure. However, the exact molecular signature of LSCs remains incompletely characterized. While it has been implicated that LSCs have a GMP-like immunophenotype, the fact that there is significant variability of outcome in LGMP-transfected mouse [20] indicates that there exists significant heterogeneity among LGMPs. Such heterogeneity was clearly recapitulated in our single-cell analysis. Our results suggest that only one subgroup of leukemic cells (Leukemia 1) is likely to be more aggressive, as validated by our functional experiments. Interestingly, our analysis shows that the gene expression profile of leukemic cells is, in general, different from HSCs, supporting the idea that the 'stemness' of LSCs is distinct from that of normal stem cells [4].

One limitation of the qPCR assay is that it is only realistic to profile a small fraction of the transcriptome. As single-cell RNA-seq technology being rapidly developed, soon it will be feasible to conduct whole transcriptome analysis in leukemic cells in a similar manner. Such analysis will be useful not only to refine the molecular signature of LSCs but also to identify critical pathways for leukemogenesis. Another important area of future research is to link the association between transcriptomic changes and genetic/epigenetic alterations. Such analysis will provide important mechanistic insights that cannot be obtained by gene expression analysis alone.

## Conclusions

Taken together, our results demonstrate that combining single-cell gene expression profiling technology and computational analyses provides novel insights into heterogeneity and cellular hierarchy in cancer. The refined characterization of the gene signature of LSCs may facilitate the development of therapeutic strategies that may overcome drug resistance, thereby improving treatment outcomes.

## Materials and methods

### Ethics statement

All animals were housed in ARCH, the animal facility of Boston Children’s Hospital, under proposals approved by the Animal Care Committee of the hospital.

### Generation of MLL-AF9 leukemic cells

Primary leukemia was generated as described before [8]. In brief, ecotropic retroviral vectors were generated by cotransfection of 293 T cells with packaging constructs. Lin-Sca1+Kit+(LSK) cells from mouse bone marrow were transduced with MLL-AF9-GFP and maintained in methylcellulose (Stem Cell Technologies, Vancouver, British Columbia, Canada) with supplemental cytokines for three days. Colonies were transplanted into sublethally irradiated (600 rad) C57BL6 recipients at 5 × 10^5^ cells per mouse. Leukemic cells were collected from bone marrow of multiple sick recipients after four weeks and then pooled for analysis.

### FACS sorting and single-cell collection

Bone marrow cells were isolated by crushing iliac crest bones, femurae and tibiae in phosphate-buffered saline containing 5% fetal calf serum and 2 mM EDTA. After red blood cell lysis, the remaining cells were stained with monoclonal antibodies, analyzed and sorted on the BD FACSAria II (BD Bioscience, San Jose, CA, USA). Individual cells were sorted directly into 96-well PCR plates loaded with PCR buffer under single-cell mode. All data were analyzed with FlowJo (Tree Star, Ashland, OR, USA).

### One tube single-cell sequence specific pre-amplification

Individual primer sets were pooled to a final concentration of 0.1 μM for each primer. Individual cells were sorted directly into 96-well PCR plates loaded with 5 μl RT-PCR master mix (2.5 μl CellsDirect reaction mix (Invitrogen, Carlsbad, CA, USA), 0.5 μl primer pool, 0.1 μl RT/Taq enzyme (Invitrogen), 1.9 μl nuclease-free water) in each well. Sorted plates were immediately frozen on dry ice. After brief centrifugation at 4°C, the plates were immediately placed on the PCR machine. Cell lyses and sequence-specific reverse transcription were performed at 50°C for 60 minutes. Then reverse transcriptase inactivation and Taq polymerase activation were achieved by heating to 95°C for 3 minutes. Subsequently, in the same tube, cDNA went through 20 cycles of sequence-specific amplification by denaturing at 95°C for 15 s, annealing and elongation at 60°C for 15 minutes.

### High-throughput microfluidic real-time PCR

Pre-amplified products were diluted five-fold prior to analysis. Amplified single-cell samples were analyzed with Universal PCR Master Mix (Applied Biosystems, Foster City, CA, USA), EvaGreen Binding Dye (Biotium, Hayward, CA, USA) and individual qPCR primers using 96.96 Dynamic Arrays on a BioMark System (Fluidigm, South San Francisco, CA, USA). Three dynamic arrays loaded with different primer sets were used for each sample plate. Ct (threshold cycle) values were calculated using the BioMark Real-Time PCR Analysis software (Fluidigm).

### *In vitro* colony forming assay

We plated 5,000 cells from each population in 1.5 ml of Methocult M3234 (Stem Cell Technologies) supplemented with IL3 (10 ng/ml), IL6 (5 ng/ml), IL7 (10 ng/ml) and stem cell factor (20 ng/ml). Methylcellulose cultures were incubated at 37°C in a humidified atmosphere with 5% CO_2_ in air. Colonies were scored on day 7.

### Computational analyses

Gene expression levels were estimated by subtracting the Ct values from the background level of 28, which approximates log_2_ gene expression levels. Ct values higher than 28 were first transformed to 28 and are represented by zero (no expression) in the data.

Unsupervised hierarchical clustering was achieved using an average linkage method and a correlation-based distance (Pearson correlation) in MATLAB. The t-SNE analysis [38] was performed using the MATLAB toolbox for dimensionality reduction [57]. This method is a variation of the stochastic neighbor embedding (SNE) method [58], which minimizes a cost function based on conditional probabilities to describe the similarities between data points in the high-dimensional space. That is, the similarity of data point *y*_*j*_ to *y*_*i*_ is estimated by the conditional probability that *y*_*i*_ would pick *y*_*j*_ as its neighbor, if neighbors were selected in proportion to their probability density under a Gaussian distribution centered at *y*_*i*_. t-SNE improves upon SNE by using a symmetrized version of the SNE cost function and a Student's t-distribution rather than a Gaussian to compute the similarity between two points, thereby making the optimization problem easier to solve. It also reduces the tendency to crowd points together in the center of the map and thus produces better visualizations [38]. For the FACS dataset, since it was computationally intractable to map all the data into a two-dimensional space (Figure 1B), we randomly sampled 5,000 non-leukemic and 5,000 leukemic cells.

SPADE analysis [41] for mapping cellular hierarchy was done in MATLAB and R. We used the 56 cell clusters that were identified by SPADE analysis of more than 1,000 cells in [20]. Each normal or leukemic cell in our dataset was then assigned to the cluster whose mean was closest to that cell based on the Euclidean distance.

A two-sided Wilcoxon-Mann-Whitney rank sum test, implemented in the coin package [59] in R, was employed to identify differentially expressed genes. *P*-values were adjusted using the Benjamini and Hochberg method [60] in R. The fold change of each gene in two cell populations was calculated as the difference of medians of the log_2_ expression levels for the two groups.

Weighted gene co-expression network analysis was done using the WGCNA package [50] in R. Anti-log_2_ transformation was applied to convert log_2_ expression levels to a normal scale. To construct unsigned weighted networks, WGCNA makes use of a power adjacency function *a*_*ij*_ = |cor(*x*_*i*_,*x*_*j*_)*|*^*β*^ to define the connection strength between any pairs of genes *x*_*i*_ and *x*_*j*_ and implements a soft power threshold (*β*) approach that aims at approximating a scale-free topology (that is, the frequency distribution, *p*(*k*), of the network connectivity, *k*, follows a power law) to the network [50,51]. For each network, we chose *β* in such a way that the model fitting index *R*^2^, defined as the square of the correlation between log_10_ (*p*(*k*)) and log_10_ (*k*), is greater than 0.85. More specifically, *β* = 6, 4, 5, and 4 for the GMP, all-leukemia, Leukemia 1 and Leukemia 2 networks, respectively. The module detection was achieved by using average linkage hierarchical clustering, which uses a dissimilarity measure based on the topological overlap matrix (TOM) [50,51], and a dynamic tree-cut algorithm. Topological overlap considers each pair of genes in relation to all other genes in the network and, as such, genes that are connected to roughly the same group of genes in the network have a high topological overlap. The modules were then visualized using a TOM plot [50,51], which is a color-coded depiction of the values of the TOM-based dissimilarity matrix. We note that all the genes were considered in constructing each of the co-expression networks in our model, but only those genes that were assigned to a co-expressed module were shown in the final networks.

## Additional files

Additional file 1: Figure S1. Distributions of different markers in the two subgroups of non-leukemic cells using FACS data. The red (blue) boxplots correspond to the non-leukemic cell subgroup given on the right (left) in Figure 1B. Additional file 2: Table S1. Single-cell gene expression data in leukemic cells. All gene expression data are represented as log_2_ expression level above the system background (Ct = 28), which is approximately equal to 28 minus raw Ct (from each qPCR reaction). Ct values higher than 28 were transformed to 28, and are represented by zero (no expression) in the data. Each column corresponds to a specific gene and each row corresponds to a single leukemic cell. Additional file 3: Table S2. List of the 33 genes used for SPADE analysis. Additional file 4: Figure S2. Prediction accuracy of the SPADE tree mapping strategy. (A) Using the 33 common genes discussed in the main text. (B) Using the average of 100 randomly selected sets of 33 genes from the data. The error bars represent standard deviation. In both graphs, the y-axis represents the fraction of cells that is mapped to a cluster within a certain distance to the original cluster. Additional file 5: Table S3. List of the identified differentially expressed genes. List of all the genes along with their adjusted *P*-values and log fold changes are given as well. Additional file 6: Table S4. List of genes in each network module represented in Figure 5. Additional file 7: Figure S3. Venn diagrams showing the overlap between the network modules given in Figure 5. Additional file 8: Text S1. Details of the differential co-expression module analysis between Leukemia 1 and Leukemia 2 using DiffCoEx. Additional file 9: Figure S4. Comparative correlation heat map showing differentially co-expressed modules between Leukemia 1 and Leukemia 2. The upper/lower diagonal of the matrix shows correlations between pairs of genes in Leukemia 1/Leukemia 2 populations. Each row and column corresponds to a gene. The modules are indicated by color bars next to the heat map. Additional file 10: Table S5. List of genes in the differentially co-expressed modules represented in Additional file 9.

## Abbreviations

AML: acute myeloid leukemia
CLP: common lymphoid progenitor
CMP: common myeloid progenitor
FACS: fluorescence-activated cell sorting
GFP: green fluorescent protein
GMP: granulocyte/monocyte progenitor
HSC: hematopoietic stem cell
IL: interleukin
LGMP: leukemic granulocyte-monocyte progenitor
LSC: leukemia stem cell
MEP: megakaryocyte/erythroid progenitor
MPP: multipotent progenitor
PCR: polymerase chain reaction
qPCR: quantitative PCR
SNE: stochastic neighbor embedding
SPADE: spanning-tree progression analysis of density-normalized events
TOM: topological overlap matrix
t-SNE: t-distributed stochastic neighbor embedding
WGCNA: weighted gene co-expression network analysis
