# Supplementary figures and images for: Characterizing heterogeneity in leukemic cells using single-cell gene expression analysis
Source: Genome Biol. 2014 Dec 3;15(12):525. doi: 10.1186/s13059-014-0525-9 (PMC4262970; doi:10.1186/s13059-014-0525-9)

Signal strength

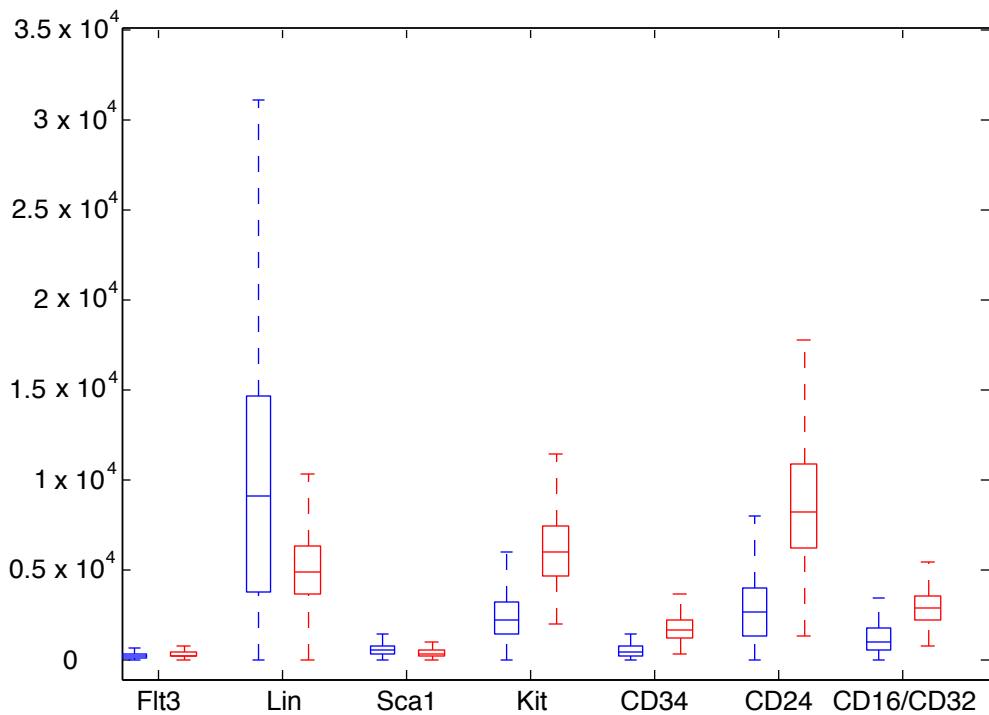

Supplement: Additional file 1: Figure S1. — Distributions of different markers in the two subgroups of non-leukemic cells using FACS data. The red (blue) boxplots correspond to the non-leukemic cell subgroup given on the right (left) in Figure 1B. [file 13059_2014_525_MOESM1_ESM.pdf]

**A**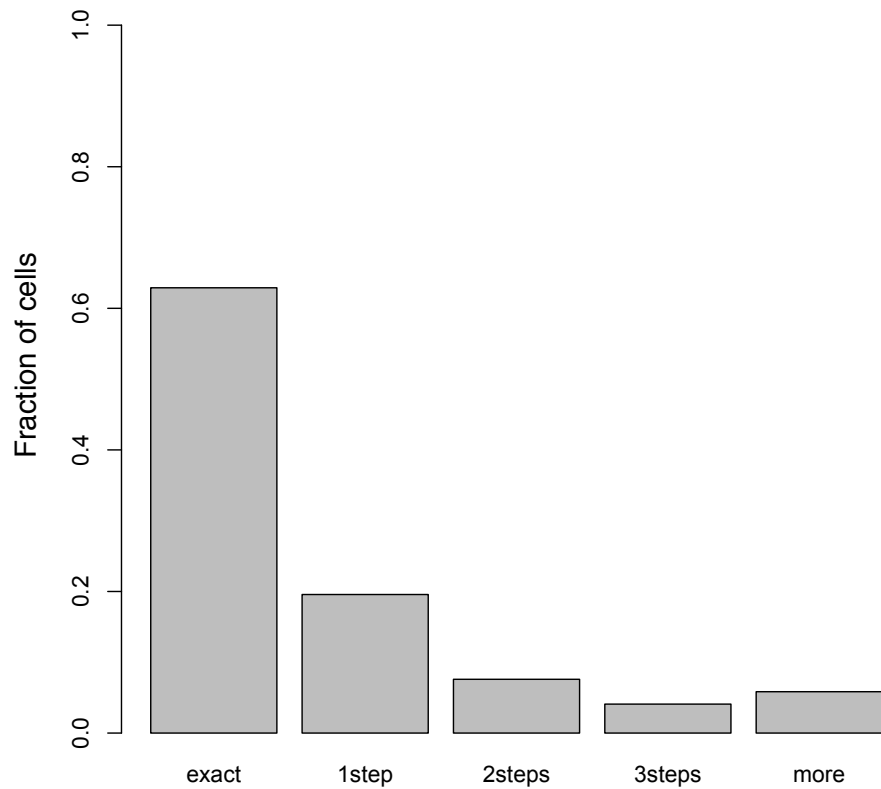**B**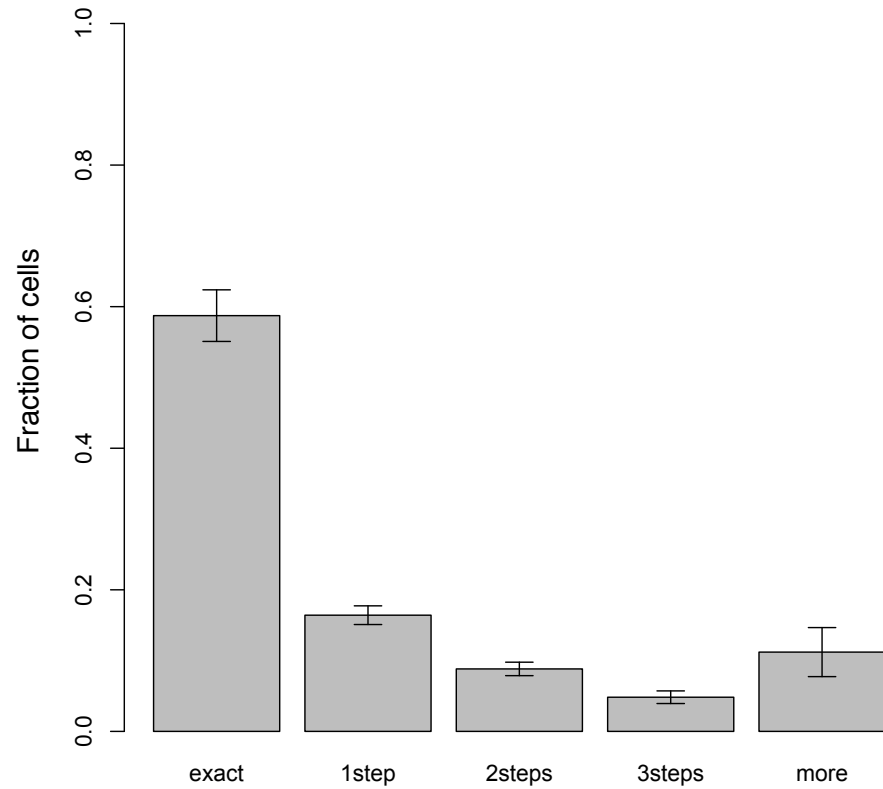

Supplement: Additional file 4: Figure S2. — Prediction accuracy of the SPADE tree mapping strategy. (A) Using the 33 common genes discussed in the main text. (B) Using the average of 100 randomly selected sets of 33 genes from the data. The error bars represent standard deviation. In both graphs, the y-axis represents the fraction of cells that is mapped to a cluster within a certain distance to the original cluster. [file 13059_2014_525_MOESM4_ESM.pdf]

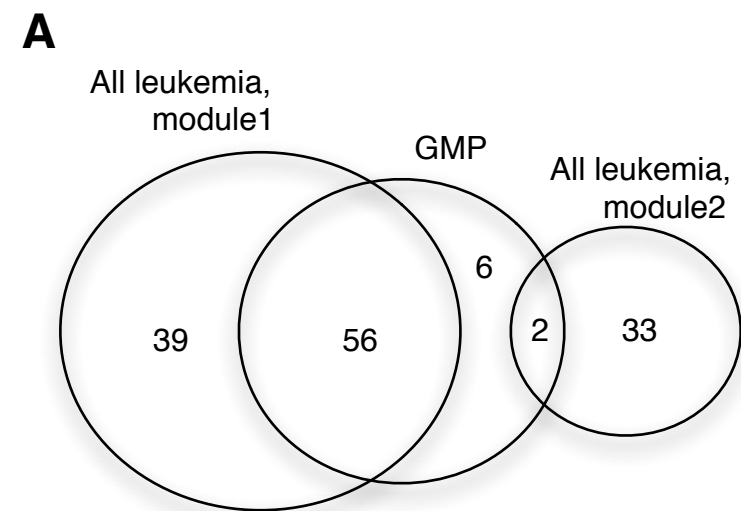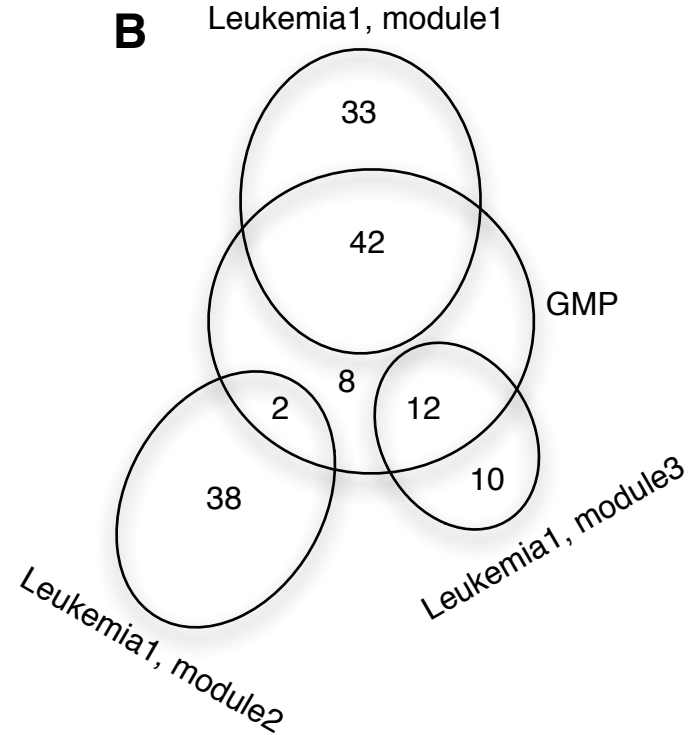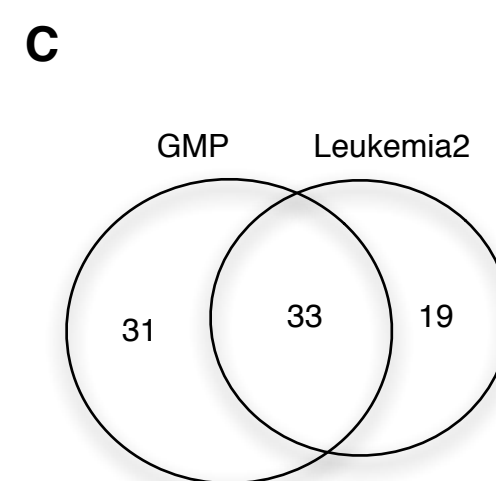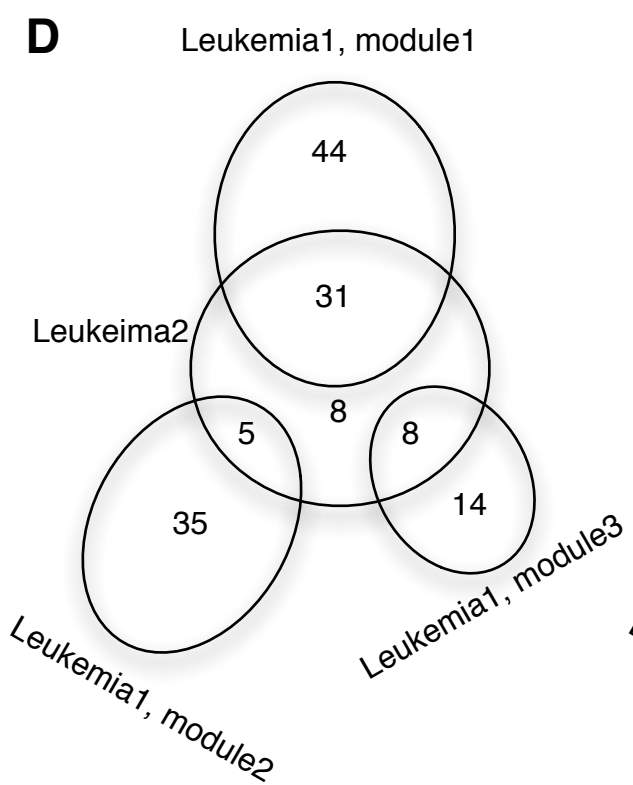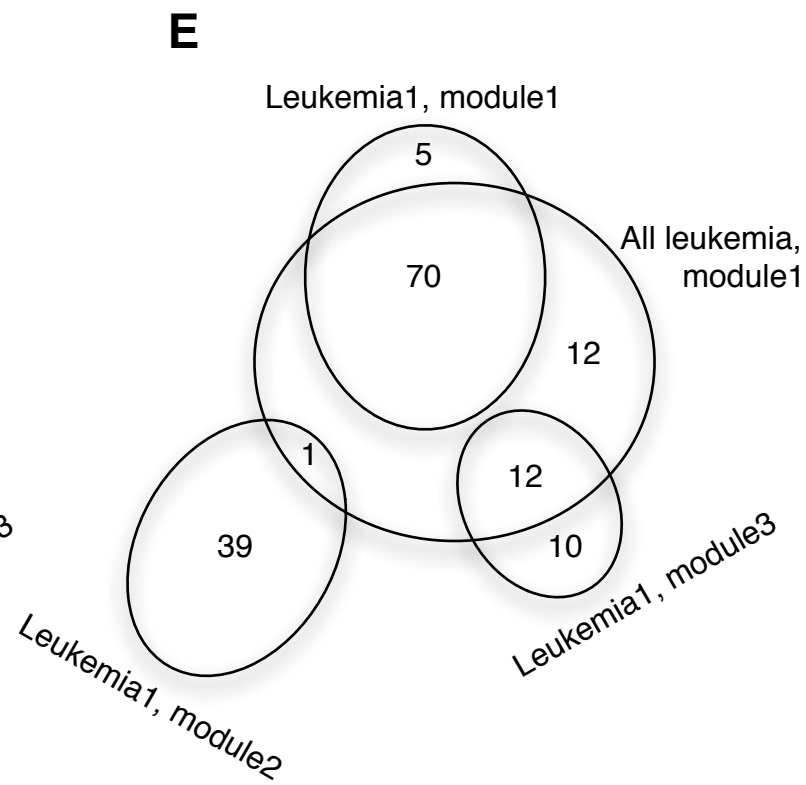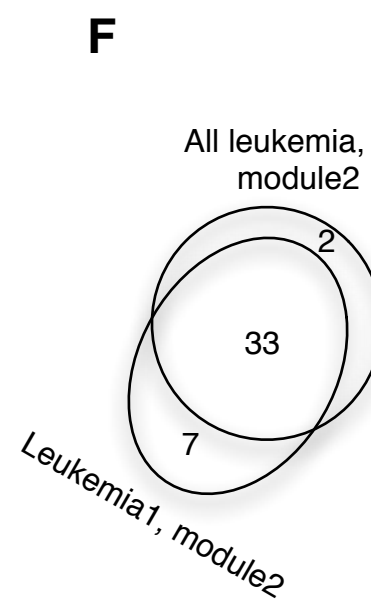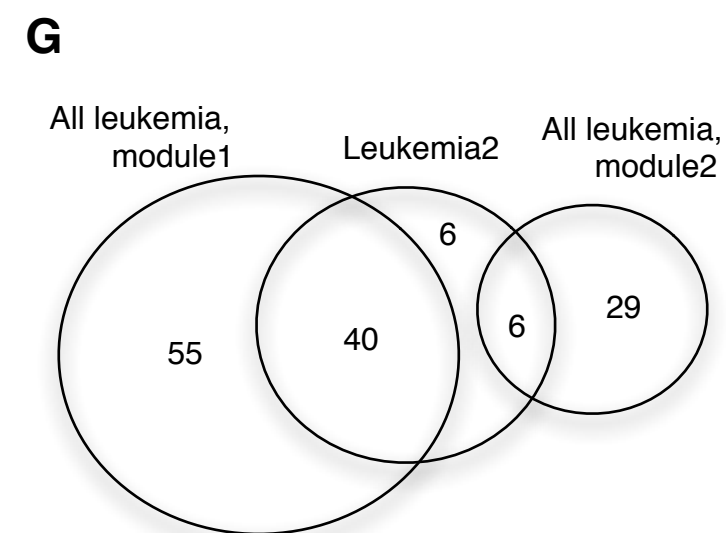

Supplement: Additional file 7: Figure S3. — Venn diagrams showing the overlap between the network modules given in Figure 5. [file 13059_2014_525_MOESM7_ESM.pdf]

Leukemia 1

Leukemia 2

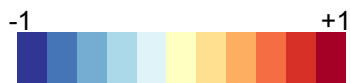

Supplement: Additional file 9: Figure S4. — Comparative correlation heat map showing differentially co-expressed modules between Leukemia 1 and Leukemia 2. The upper/lower diagonal of the matrix shows correlations between pairs of genes in Leukemia 1/Leukemia 2 populations. Each row and column corresponds to a gene. The modules are indicated by color bars next to the heat map. [file 13059_2014_525_MOESM9_ESM.pdf]
