# Supplementary material for: Characterizing heterogeneity in leukemic cells using single-cell gene expression analysis
Source: Genome Biol. 2014 Dec 3;15(12):525. doi: 10.1186/s13059-014-0525-9 (PMC4262970; doi:10.1186/s13059-014-0525-9)
Supplement: Additional file 8: Text S1. — Details of the differential co-expression module analysis between Leukemia 1 and Leukemia 2 using DiffCoEx. [file 13059_2014_525_MOESM8_ESM.pdf]

Supplementary text for "Characterizing heterogeneity in leukemic cells using single-cell gene expression analysis"

Assieh Saadatpour, Guoji Guo, Stuart H. Orkin, Guo-Cheng Yuan

Details of the differential co-expression module analysis between Leukemia 1 and Leukemia 2 using DiffCoEx

To further identify differentially co-expressed modules between Leukemia 1 and Leukemia 2 cell subtypes, we utilized the DiffCoEx method implemented in R (Tesson et al., 2010). This method is built upon WGCNA (Langfelder and Horvath, 2008; Zhang and Horvath, 2005) and demonstrates the differentially co-expressed modules between two conditions in a comparative heat map. In this method, first the correlation change between two conditions (here, Leukemia 1 vs. Leukemia 2) is quantified as the difference between signed squared correlation coefficients. Then a soft power threshold (here, β=4) is used to transform the correlation values so that the weight of large correlation differences is emphasized compared to lower, less meaningful, differences. The differential modules are then identified using hierarchical clustering with a dissimilarity measure based on the topological overlap matrix (Langfelder and Horvath, 2008; Zhang and Horvath, 2005) and a dynamic tree-cut algorithm. Finally, the modules are visualized using a comparative correlation heat map based on the signed squared correlation coefficients. Here we first applied the anti-$\log_2$ function to the data before performing DiffCoEx analysis.

This method identifies two differentially co-expressed modules between Leukemia 1 and Leukemia 2 (Additional file 9: Figure S4 and Additional file 10: Table S5), with only the orange module associated with strong difference in co-expression patterns. This is justified in part by the fact that the difference between the average values of the upper and lower diagonal entries in the comparative correlation heat map (Additional file 9: Figure S4) for the orange module is much higher than that for the purple module (0.23 vs. 0.08). Of the 85 genes in the purple module, 76 (89%) are in Module 1 of the all-leukemia network (retained in both leukemia 1 and 2 networks) that we reported in Figure 5 using the WGCNA analysis, while it has no overlap with Module 2 of the all-leukemia network. Interestingly, the orange module has only 8 genes (13%) overlap with Module 1 of the all-leukemia network, while it has 35 genes (56%) common to Module 2 of the all-leukemia network. In addition, all the 14 differentially expressed genes between Leukemia 1 and 2 are present in the purple module, which may be associated with the lack of differential co-expression.

References

Langfelder P, Horvath S: WGCNA: an R package for weighted correlation network analysis. *BMC Bioinformatics* 2008, 9:559.

Tesson BM, Breitling R, Jansen RC: DiffCoEx: a simple and sensitive method to find differentially coexpressed gene modules. *BMC Bioinformatics* 2010, 11:497.

Zhang B, Horvath S: A general framework for weighted gene co-expression network analysis. *Stat Appl Genet Mol Biol* 2005, 4(1):Article17.
